# How men and women learn about sex: multi-generational perspectives on insufficient preparedness and prevailing gender norms in Scotland

**DOI:** 10.1080/14681811.2019.1683534

**Published:** 2019-11-07

**Authors:** Susan Patterson, Lisa McDaid, Kate Hunt, Shona Hilton, Paul Flowers, Lesley McMillan, Dona Milne, Karen Lorimer

**Affiliations:** a MRC/CSO Social and Public Health Sciences Unit, University of Glasgow, Glasgow, UK; b Institute for Social Marketing, Faculty of Health Sciences and Sport, University of Stirling, Stirling, UK; c Department of Social Sciences, Glasgow School for Business and Society, Glasgow Caledonian University, Glasgow, UK; d Public Health, NHS Fife, Fife, UK; e School of Health & Life Sciences, Glasgow Caledonian University, Glasgow, UK

**Keywords:** Sexual health, sex education, school, gender norms, life course, young people, relationships

## Abstract

Attitudes towards sexual health and relationships are learned from a young age, and there is an ongoing need for innovative and comprehensive approaches to sex education that keep pace with rapidly changing contexts of people’s lives. We used thematic analysis of data from two qualitative studies in Scotland to explore learning contexts from a multi-generational perspective, as well as the influence of different socio-cultural factors on provision, access to and experience of sex education. The importance, but inadequacy, of school as a source of learning, was a persistent theme over time. Participants’ strategies to address perceived gaps in knowledge included experience, conversations, vicarious and online learning. Gender and age differences emerged, with younger participants more likely to go online for information, and prevailing gender norms shaping attitudes and behaviours across both study groups. Participants who identified as gay, lesbian or bisexual described feeling particularly unprepared for sex and relationships due to the narrow, heteronormative content received. Although schools continue to be a common source of information, it appears that they fail to equip young people for their post-school sexual life-course. We recommend the mandatory provision of comprehensive, positive, inclusive and skills-based learning to improve people’s chances of forming and building healthy, positive relationships across the lifespan.

## Introduction

Attitudes towards sexual health and relationships are learned from a young age, and people draw on a wide range of sources that can influence these. Good quality sex and relationships education in the form of education that is age-appropriate, comprehensive, sex-positive, inclusive, culturally relevant, and competently delivered, is considered important in helping people to have safe and enjoyable sex lives (Pound et al. 2017). Research has however highlighted disparities in the quality and content of sexual health and relationships education: topics such as relationships, sexuality and same-sex relationships are not mandatory, and there is inconsistency in implementing guidance within schools and local authorities resulting in substantial variety in the sex and relationships education that young people receive (Ofsted 2013; Bailey et al. 2015). Recently, the UK government announced plans to make relationships and sex education statutory in all secondary schools in England and Wales, and relationship education statutory in primary schools. In Scotland, comprehensive sexual health and relationships education has yet to be made compulsory, but the Scottish Government has announced that teaching on lesbian, gay, bisexual, transgender and intersex (LGBTI) rights is to be embedded across the school curricula (https://www.gov.scot/news/lgbti-education/) and there is work underway nationally to develop comprehensive materials for sexual health and relationships education in Scotland .

Previous research has noted that young people typically rely on ‘traditional’ educational sources, such as schools (Alldred and David 2007; Parker 2014; Tanton et al. 2015), in their learning about sex and relationships, and they are an important environment for preparing young people for their future sexual lives (Grose, Grabe, and Kohfeldt 2014). It is important to equip young people in school with skills for both immediate and later sexual decision-making to facilitate safe and fulfilling sex lives and healthy relationships as they move through life (Bourke et al. 2014). In recent years, digital technologies have become increasingly important as a source of sexual health information, presenting both opportunities and challenges for people seeking sexual health information and services (Pound et al. 2017). Given the highly dynamic information landscape that we live within, we need to maintain up-to-date understandings of the contexts and sources of information that influence learning, knowledge, skills, expectations and norms around sexual health and wellbeing. There is a particular need for research that documents these changing means of sexual health learning and determines the extent to which sources meet information and learning needs (Tanton et al. 2015). As learning contexts, and the influence of different socio-cultural contexts on provision, access to and experience of sexual health education have changed over time, we sought to explore multi-generational perspectives on sexual health learning, combining qualitative data from two studies: the Deprivation, Masculinities and Sexual Health (DeMaSH) study (men and women aged 18–40), and the Young People’s Online Sexual Health Information Study (OSHI) (men and women aged 16–19). Understanding if, and how, sexual health and relationships education in schools prepares young people for the realities of sex and relationships has potential implications for schools, health services, policy makers, and future research.

## Methods

Recruitment and data collection methods for each of the two studies drawn upon here are detailed below, followed by a description of the subsequent data synthesis and our approach to integrating findings.

### Deprivation, masculinities and sexual health study (DeMaSH)

In the DeMaSH study, we conducted face-to-face, individual, in-depth interviews with 19 men and 16 women aged 18–40 years (who identified as heterosexual) between February 2014 and April 2015. Participants resided in areas classified as the 10% most economically and socially deprived, across Glasgow, Edinburgh, Dundee and three areas of the Highlands, using the Scottish Index for Multiple Deprivation (SIMD). Participants were recruited from various community-based organisations (see Table 1 for participant characteristics). In this paper, we primarily focus on responses prompted by the opening interview question, ‘How did you learn about sex?’. Further details on recruitment, data collection and additional topics explored (e.g. experiences of living/growing up in the area; their views about the lives and expectations of men and women in their local areas; and their sexual health understandings, knowledge and behaviours) are described elsewhere (Lorimer et al. 2018). Ethical approval was obtained from Glasgow Caledonian University, School of Health and Life Sciences Research Ethics Committee (application no: SYEC12/APP202). Informed consent to audio record interviews was also obtained.

**Table 1.** Characteristics of participants in one-to-one interviews.

Characteristics	*N*	%
Sex		
Male	19	54.3
Female	16	45.7
Age		
18–25	16	45.7
26–40	19	54.3
Highest educational qualification		
Undergraduate degree	1	2.9
Scottish Higher	9	25.7
Vocational/apprenticeship	2	5.7
Scottish ‘standard grade’	17	48.6
None	6	17.1
Marital status		
Single	17	48.6
Long-term relationship/married	16	45.7
Other	2	5.7

### Young people’s online sexual health information study (OSHI)

The OSHI study included paired qualitative interviews with 49 women and men (aged 16–19), exploring perspectives on and experiences of sexual health information contexts (e.g. school, friends), with a focus on the online environment (Martin 2017). Young people were recruited between January and August 2015, using a range of recruitment strategies, including through youth and community organisations, posters, social media posts and snowball sampling. Participants were recruited across Scotland from areas that varied by urban/rural classification (as defined by the Scottish Government 2014) and by economic and social deprivation (as measured using SIMD). In total, 30 women and 19 men (aged 16–19), diverse in gender, sexuality, religion and geographical location (see Table 2) took part in 25 interviews (this included two individual and one triad interview necessitated by circumstances rather than participants’ preferences). In terms of their relationships with one another, most participants described themselves as ‘best friends’, or childhood/school friends, while two pairs were related to each other, and two pairs described themselves as being acquaintances. Interviews were conducted face-to-face using a semi-structured interview schedule which explored participants’ perceptions of, and experiences relating to, different sources of sexual health information, and their sexual health understandings, attitudes and behaviours. Ethical approval was obtained from the University of Glasgow, College of Social Sciences Research Ethics Committee (application no: 400,140,170). Informed consent to audio-record the interviews was obtained.

**Table 2.** Participant characteristics.

Participants characteristics	*N*	%
Sex		
Male	19	38.8
Female	30	61.2
Age		
16	16	32.7
17	15	30.6
18	9	18.4
19	9	18.4
Education/work status		
Still at or recently left school	22	44.9
Attending College or University	14	28.6
Employed	5	10.2
Looking for work	3	6.1
Not provided	5	10.2
Sexuality		
Heterosexual	38	77.6
Gay/lesbian	5	10.2
Bisexual	3	6.1
Prefer not to answer	3	6.1
Religion		
Roman Catholic	10	20.4
Other Christian	2	4.1
Other	37	75.5
Locality		
Large urban area	20	40.8
Small u﻿rban area	15	30.6
Accessible small town	8	16.3
Accessible rural town	6	12.2
Area deprivation		
15% most deprived areas	11	22.4
Other	38	77.6

### Analysis, synthesis and integration of data

Audio-recorded interview data from both studies were transcribed verbatim and anonymised (using pseudonyms). Data were imported into QSR NVivo 10 software to support rigorous analysis that involved two stages: i) thematic analysis of interview data from each study using framework; and ii) synthesis of key findings and data integration. The framework approach to analysis was adopted to ensure a systematic synthesis of key interpretations and themes across the data (Ritchie and Spencer 2002). This involves five key stages: familiarisation; identification of a thematic framework; indexing; charting; and mapping and interpretation. The key thematic findings identified at the charting stage from the DeMaSH and OSHI studies were then combined for mapping and interpretation, to facilitate comparison and synthesis of findings generated across the two studies. The table was organised to allow consideration of the findings related to specific learning contexts and themes. Throughout the analysis process, emergent themes were discussed, and consensus reached after discussion of ambiguities by SP, KL and LMcD.

## Results

The findings presented below focus on how participants described learning about sex, including reflections of school-based sex education and how gaps in education were filled using other sources.

### Reflections of school-based sexual health and relationships education

School-based sexual health education emerged from both study groups as an important source of learning about sex and sexual health, which given the different age range, social and regional contexts of the two studies, suggests some continuity in the importance of school as a source of sexual health learning. It seems that even within a rapidly evolving information landscape, school remains an important and desired source of sexual health learning for young people (Alldred and David 2007; Parker 2014). However, participants’ satisfaction and evaluations of their experiences did not vary substantially across the age range, with most describing school-based sexual health and relationships education as leaving them feeling unprepared for positive relationships and good sexual health:

To be honest, the school didn’t even teach you very much. Like, you done it [sexual health and relationships education] in like third year [age 13-15] and you done it like a few weeks on it once a week and that was about it. That’s all I can remember getting at school, they don’t really give you much at all … information. I think they just, kind of, expect you to know after that few weeks, but they don’t actually tell you much. (Melissa, 16, OSHI)

You know, ‘cause you learnt a wee bit, but wouldnae really – didn’t really learn nothing at school, really. You know, I learnt it myself, basically. (Scott, 40, DeMaSH)

The above quotes demonstrate that participants expressed a similar lack of satisfaction and preparedness, despite some reflecting on sexual health and relationships education they had received over twenty years previously. This highlights a lack of progress regarding the quality of sexual health and relationships education provision in Scotland, and the continued failure to provide young people with comprehensive sex education, which is also demonstrated within the wider UK context (Bailey et al. 2015), and in the USA (Pound, Langford, and Campbell 2016).

Participants typically described school-based sexual health and relationships education as focusing on a narrow range of topics, particularly prevention (e.g. contraception) and outcomes of risk behaviours (e.g. unwanted pregnancy and STIs), but without providing the practical information necessary to mitigate such risks. Accounts illustrated shortcomings in practical teaching around safe sex, using contraception, and accessing STI treatment. For example, Cleo (19, OSHI) recounted not learning how to use condoms: ‘we learned about STDs which was good to be aware of … but other than that, you didn’t know like how to say use a condom and stuff like that’.

Few participants mentioned anything positive when describing their recollections of school-based sexual health and relationships education. A small number of participants within the OSHI study commented specifically on the lack of teaching about broader and more pleasurable aspects of sex. For example, Nicola and Ralph noted the absence of discussion of masturbation:

Nicola (17, OSHI): Well they’ve [schools] not been letting young people know, like, they should talk to them like face to face tae show that, like, it’s okay if you have these questions, it’s okay tae do this, it’s perfectly normal. But they don’t talk about, like, a lotta things like masturbation or whatever, like they don’t talk about that because, like … I don’t know why because, like, a lotta kids do it and they feel like horrible for doing it …

Ralph (19, OSHI): It should be talked about …

Nicola: You know “Its fine, it’s okay. Don’t worry” …

The quote also suggests that taboos around subjects such as masturbation could be tacitly reproduced, causing feelings of shame. Research has highlighted how infrequent, negative and moralistic teaching that fails to discuss sexual issues openly can contribute to stigma and anxiety, reproducing perceptions of sex as a ‘taboo’ that should not be talked about or prepared for (Bay-Cheng 2001; McKee, Watson, and Dore 2014; Woodcock, Stenner, and Ingham 1992). This study highlights the continued focus on messages of sexual ill-health and risk, over sexual-wellbeing and pleasure, and provision of practical information, emphasising the role of school in perhaps unintentionally reinforcing a moralistic and negative culture around sexual health.

Participants across both study groups highlighted that school-based sexual health and relationships education did not sufficiently cover the realities of sex and relationships, including aspects of communication and broader wellbeing. Illustrating this, some participants recalled being shown educational videos, which they found unenlightening. For example, Sinead (19, OSHI) recalled being shown: ‘wee stupid videos, like the people walk into a room, then shut the door, and then come out like, ‘hmm … what did we just dae?’, while Tim (29, DeMaSH) recalled being shown a video about sex featuring rabbits, which he said failed to ‘show you, like, the interaction’. Participants also expressed an awareness that ‘realistic’ sexual encounters can be more awkward and unpleasant than the sanitised depictions in educational videos, although it is unclear from where such ‘realistic’ representations may stem, given the views towards pornography use we detail in the next section. Kayleigh (19, DeMaSH) felt that her sex education did not prepare her for the awkward and ‘not always very nice’ physical aspects of sex. Beyond the need for practical education about the mechanics and realities of sexual encounters, Luke (21, DeMaSH) highlighted the need for young people to be taught to cope with emotions they may experience after sex:

Sometimes when you’ve slept wi’ someone, after you might, like, you feel a bit shit, you get a bit anxious … ’oh fuck, what did I dae [do] that for?’ […] I’m sure a lot o’ girls after they sleep wi’ guys and then they kick them out the door at one o’clock or random times in the morning, they feel awful. You need tae learn how tae deal wi’ it, cope wi’ it, stuff like that. (Luke, 21, DeMaSH)

This quote emphasises the need for a holistic approach to sexual health and a focus on developing skills to deal with emotional aspects of sex (Lohan et al. 2018; McMichael and Gifford 2010).

Our research also highlights how sexual health and relationships education still appears to fail many young people by undermining, subtly if not explicitly, non-heterosexual relationships. Within the OSHI study, participants who identified as gay, lesbian or bisexual, described feeling particularly disadvantaged and unprepared due to the narrow and heteronormative content provided. Abbie (16, OSHI) critiqued her school’s lack of information provision for gay, trans and non-binary people, while Liam (16, OSHI) observed that his school provided no positive, practical information about anal sex in heterosexual or gay relationships:

I think it’s quite ironic when we were doing STIs, obviously one o’ the big ones was HIV/AIDS an’ they were saying that that’s more commonly transmitted, like, through the likes of anal sex, but you [sic.] never actually taught us about anal sex. Like you know, like the safety side, using lubricant, whatever. An’ it’s like if you’re going to tell us “Here’s the dangers”, at least tell us how to prevent it. (Liam, 16, OSHI)

This account illustrates that by failing to provide relevant information, education can neglect non-heterosexual relationships, and other forms of penetrative sex. Research has highlighted that the heteronormative narratives embedded within school-based sex education underprepare young people for sexual relationships and may contribute to prejudice and stigma (Fornby 2011). Comparing our findings with those of Buston and Hart (2001), it seems little has changed in Scottish schools in almost 20 years. Elsewhere, it has been reported that trans and non-binary youth could find school sex education similarly excluding (Riggs and Bartholomaeus 2018). It is also important to note, that by narrowing the focus to vaginal penetrative sex, schools seem to be failing to prepare young people in heterosexual relationships for a variety of sexual behaviours, which could impact on both disease prevention, but also on pleasurable and satisfying sexual experiences. Research has shown that anal sex is increasingly prevalent among young men and women (Mercer et al. 2013; Chandra, Mosher, and Copen et al. 2011), but largely neglected as part of school-based sex education (Marston and Lewis 2014), leading to calls for ‘an urgent need for harm reduction efforts targeting anal sex to help encourage discussion about mutuality and consent, reduce risky painful techniques and challenge views that normalise coercion’ (Marston and Lewis 2014, 1).

### Filling gaps using other sources: learning ‘by doing’, pornography and sharing experiences with friends

Young people’s sexual education in school may be a driver for information seeking from other sources, with participants describing purposively addressing gaps in sexual health and relationships education content via experiential, conversational and vicarious learning, to better prepare for practicalities of sex and sexual relationships. Participants, particularly within the older (and therefore more likely sexually experienced) DeMaSH study, described learning about sex experientially and through ‘experimentation’. For example, Scott (40, DeMaSH) described learning ‘by doing’, and declared: ‘you just – practice makes perfect, really, don’t it? You know. That’s how I’ve learnt’. In both studies, participants tended to think that men would be more likely to learn about sex experientially, while women may learn from other sources:

They [women] might learn more about sex from a girlfriend, telling them how they had sex with another guy. I think. Or from a book. Because, uh, which is different from guys, we don’t learn about sex from that. We just want to do it. (Shane, 33, DeMaSH).

Men in the DeMaSH study tended to believe women gained knowledge about sex and relationships through romantic literature and film, as well as from conversations with female friends. In reality, women in the DeMaSH study were almost as likely as men to say they learned through experience, although they did also offer additional sources such as magazines and conversations with friends.

Across both study groups, gender differences were evident in both the likelihood of discussing sex with friends, and how supportive and earnest those discussions were likely to be. Female participants often described learning from friends as part of a wider practice of mutual emotional support, exemplified by Laura and Courtney:

Laura (16, OSHI): We talk about everythingCourtney (17, OSHI): Yeah we doLaura: If we don’t know something, we’ll help each other outCourtney: Try and figure it out

In contrast, male participants tended to describe discussing sex with male friends in flippant or boastful terms, with conversations comprising performances of masculine prowess rather than earnest exchanges of support. Jacob and Connor (18, OSHI) both explained that they would never talk in a ‘serious’ way about sex with their male friends, while Declan said that men often talk with other men about sex, but not in a manner conducive to learning:

Mates are mates aren’t they. They’ll tell them anything. Like, if they end up … like, sleeping wi’ a nice lassie or something they’re bound tae comment an’ start mouthing aff aboot [off about] it. So I don’t know if I’d say that I learned anything fae [from] my mates. I mean, I get told a lot from my mates. (Declan, 20, DeMaSH)

Our finding that female participants were more likely to value drawing on friends for emotional and informational support, highlights gendered communication norms as a potential barrier to young men talking positively and constructively about sexual health with their friends. Some participants indicated that men’s reluctance to discuss certain aspects of sex openly may be due to stigma about sexual inexperience. For example, Darren (17, OSHI) stated that he would be ‘too ashamed’ to admit to a friend that he did not know about sex, before going on to clarify that he ‘did’ know about sex. Previous research suggests that young men are expected to be sexually knowledgeable (Buston and Wight 2006; Limmer 2010), and it is likely that, for young men, being seen to be experienced or maintaining a façade of jovial indifference about sexual health is a performative means of protecting masculine social status. The power of such pressures and the related incentive to exaggerate sexual experience could conceivably compromise young men’s preparedness for healthy sexual behaviours and relationships. However, some male participants exhibited a critical awareness of these masculine norms. For example, Aiden (29, DeMaSH) described men as ‘bravado orientated’ and described ‘old stereotypes’ of men not discussing their thoughts and feelings (and sexuality) as ingrained, particularly amongst men in the area of the city he had grown up in, and disappointingly this still seems to exist, as we can see from the OSHI study participants.

Additionally, male participants across both studies were more likely to describe using pornography to learn about sexual norms and women’s bodies, echoing findings from previous literature (Buston and Wight 2006; Scarcelli 2015; Albury 2015; Parker et al, 2014; Tanton et al. 2015). For example, when asked about learning about sex, Luke (21, DeMaSH) described learning about sex positions predominantly from pornography rather than from school, explaining ‘ … when you get sex education at school you don’t actually ever see any sex, if that makes sense’. This perspective was reinforced from other participants’ criticism that depictions of sex in school-based sexual health and relationships education were too abstract to be of practical use and identified this as one reason why pornography might be used in an attempt to fill a perceived ‘gap’ in knowledge.

Female participants within both studies were aware of their male peers’ use of pornography, but typically expressed less interest in it themselves. For example, Emma said:

I dinnae really ken [don’t really know] much lassies [women] that are intae [into] like porn and stuff like that but, like, pretty much every boy that I ken [know] has at least watched it once, and, like, they all have like photies of women wi’ their boobs out and stuff, so I think that’s a big influence for males, specifically. (Emma, 17, OSHI)

Although female researchers conducted the interviews for both studies, it is possible that female participants were reluctant to talk about their own pornography use due to possible stigma and perceived gender norms around ‘appropriate’ femininity. Gender differences in attitudes are likely symptomatic of wider, culturally ingrained gendered perceptions of pornography, which is continually presented as more acceptable for men than women (McKee, Albury, and Lumby 2008; Scarcelli 2015). Whether women used pornography or not, many expressed strong views against it, particularly in relation to concerns about generating unrealistic expectations of sex, disrespectful perceptions of women and potentially sexually coercive behaviours by men. For example, Emma (32, DeMaSH) worried that pornography made men ‘expect more from sex’, while Jodie (29, DeMaSH) worried that pornography caused men to view women as ‘like pieces of meat’. Some male participants also expressed criticism of pornography as an educational resource, indicating their recognition that it is ‘fantasy sex’ rather than a ‘healthy kind of sex’ (Shane, 33, DeMaSH). Indeed, while Joe (16, OSHI) explained he had learned about sex from pornography, he also said that sex as portrayed in pornography is ‘nothing like what sex is like in real life’. Thus, some male participants did critically engage with the dissonance between pornography and ‘real-life’ sexual encounters, providing some evidence of awareness of the limits of pornography as a learning source. Nonetheless, the continuing (or rather sole) use of pornography as a source of learning, particularly amongst men, is concerning, as it largely reinforces objectification and submission of women (Klaassen and Peter 2015) and prevailing gender norms about sex and hegemonic masculinity (Tylka 2015; Peter and Valkenburg 2016). It is particularly disappointing to see the persistence of these views over time.

### The role of online technologies in changing learning practices

In recent years, digital technologies have become important as a source of sexual health information, presenting opportunities and challenges for people seeking sexual health information and services (Pound et al. 2017), especially young people (Tanton et al. 2015). Within this study, amongst the younger OSHI participants, online technologies were identified as a key source of sexual health learning, with most citing the Internet as their main source of sexual health information currently, and a ‘natural’, ‘obvious’ or ‘automatic’ primary resource, particularly for teenagers. Typical responses to the question ‘What would you do if you were looking for information or advice about sexual health?’ included ‘yeah I always go to the internet’ (Claire, 17, OSHI) and ‘just Google it usually’ (Jacob, 18, OSHI). This is corroborated by findings from the Third National Survey of Sexual Attitudes and Lifestyles (Natsal-3), which found that younger people, and those who reported higher risk sexual behaviours, were more likely to have recently used the Internet to access information and support for sexual health (Aicken et al. 2016).

Participants in the OSHI study primarily described using the internet to seek particular sexual health information in response to specific concerns, for example, about contraceptive use or STI symptoms. Those who struggled with face-to-face communication about sexual health discussed the convenience, accessibility and anonymity of the internet in comparison to traditional offline sources. Amelia (17, OSHI) described saving ‘weird’ topics for the Internet, while Rowan (16, OSHI) valued the freedom of being able to receive anonymous advice online and typified the perception of the online context as safe from judgement, unlike face-to-face encounters. Some also described using the internet to ‘self-teach’, to confirm or expand general sexual health knowledge, which they felt had not been sufficiently covered in school (although no participant discussed being taught to negotiate online relationships or how to seek sexual health information online within their school-based sexual health and relationships education). For example, Connor (18, OSHI) recalled accessing Wikipedia to supplement sexual health and relationships education classes, while Liam encapsulated how inadequate teaching about sexual health and relationships in school can drive online self-teaching:

So I find that, you know, my knowledge is like reading online, like going on to like you know Pasante’s or Durex, whatever their websites. That’s mainly how I’ve got my knowledge … more just self-taught myself ‘cause the school couldn’t do that apparently … (Liam, 16, OSHI)

Such accounts support Simon and Daneback (2013) hypothesis that poor-quality school-based sex education may ‘open the door for emerging technologies to serve as resources for sexual script building’ (p.305). Our study suggests that this could particularly be the case for those who identified as gay, lesbian and bisexual, some of whom described self-teaching with online information as a counterbalance to the narrow content of traditional sex education.

In contrast, few participants from the DeMaSH study discussed the internet as a source of learning, and consistently referred to learning about sex from school, friends and through experience. Although not specifically probed, it remains striking that no DeMaSH participant mentioned use of the Internet to gain information, and instead typically characterised the Internet as a potential risk rather than a learning resource. For example, some DeMaSH participants discussed their concerns about the internet making pornography more accessible:

Like online and stuff. I mean, we didn’t have internet when I was growing up either, so … Yeah. So never accessed porn or anything like that.

Int: So when you say things are so accessible, what sorts of things do you mean?

I think probably porn is really accessible for kids. Quite worryingly so. And just in general, pop music, the lyrics. How they dress in music videos, things like that. And it’s just really, really acceptable (Angela, 34, DeMaSH).

Public concerns about young people’s online safety are well established, particularly in relation to ‘grooming’ and exposure to, and creation of, sexually explicit content (Livingstone et al. 2014). Only a minority of DeMaSH participants described the internet as a positive source of learning for young people:

It’s certainly a lot easier tae access porn an’ easier tae access stuff about sexual health an’ stuff as well withoot … if, obviously, you think there’s something wrong an’ you don’t want tae go tae your doctors, do you know what I mean? You can research it online. Stuff like that. (Tom, 32, DeMaSH)

Tom highlighted both the risks and opportunities that he considered to be presented by online technologies for sexual health learning. Daniel (38, DeMaSH) hinted that he would take the opportunity to learn online if he had been younger: ‘Internet, nowadays, innit. See, if I was young nowadays, I would learn by the Internet, probably’.

Our study has highlighted how the role of the internet in the sexual health information landscape has grown over time, which presents huge opportunities, but also challenges (see Patterson et al. 2019). Yet, despite the important role of online technologies in most people’s lives, accounts of sexual health and relationships education from DeMaSH and younger OSHI men and women suggested that class content contained little recognition or integration of the online context in class-based sexual health and relationships education. This highlights the need for schools to keep pace with the contexts that are relevant to young people, and include content in classes on effectively and safely negotiating online environments to minimise risk, and maximise pleasure and wellbeing. Teaching digital literacy and media/porn literacy should be encouraged to prepare young people to navigate such information sources (Albury 2015; Elwick et al. 2013).

## Discussion and conclusions

This study has several strengths, notably the availability of two rich sources of qualitative data, focusing specifically on sexual health collected at similar times, which allowed analysis of experience and sources of sexual health learning spanning more than two decades. It provides insights into a diverse range of experiences and practices of participants from across contexts, and beyond school and clinical settings in which much sexual health research is conducted. The study methodology also has limitations. All participants’ accounts are retrospective, particularly for the DeMaSH participants, and it is important to note that the data are participants’ own narratives and representations of their experiences, perceptions of which could change over time. While participants were diverse, some groups were not represented, particularly people from minority ethnic communities and, in the OSHI study, those from remote, rural locations.

The participants who took part may have been self-selecting for a willingness to discuss sex and sexual health, and, in the case of the OSHI study, of having a social support network to draw on for the paired interview. As the OSHI study recruited young people mainly in existing friendship pairs, this study group may have underrepresented those without such social support, and who may struggle most to communicate about sexual health. Furthermore, the dynamics of pairs’ social relationships were apparent within the OSHI interviews. While most selected close friends to be interviewed with, dynamics and power relationships within pairs varied, and in some pairs one participant dominated, particularly where relationships were characterised as short-term acquaintanceship rather than long-term friendship. It is important to note that DeMaSH participants were asked an initial open question about how they learned about sex, with no specific prompt about online learning, while conversely OSHI participants were recruited to a study with a particular focus on the online environment. This could have prompted greater reflection on the topic in the OSHI study. Similarly, participants were not asked about online sexual harassment, online bullying or sexting, all of which research has suggested require greater focus in schools (Jørgensen et al. 2019).

Nevertheless, this paper provides new insights into the sexual health learning contexts experienced by women and men from diverse age groups and backgrounds in Scotland. Gender and age differences emerged, with younger participants more likely to go online for information, and prevailing gender norms shaping attitudes and information practices across both study groups. We found that school remains a commonly reported and important source of learning about sex, but formal sexual health and relationships education still leaves young people feeling unprepared for their future sexual lives and the realities of sex and building positive relationships. Our findings also suggest that inadequate teaching in schools may drive some young people to seek sexual health information in other ways, including through pornography and increasingly online, but that schools do not seem to prepare young people to effectively navigate these sources and environments to both avoid risk, and to facilitate positive intimacy and pleasure. Thus, this study highlights continuing gaps in formal sexual health education in Scotland.

Our findings broadly align with those of research conducted in other similar policy contexts (Pound, Langford, and Campbell 2016; Fox, Hale, and Gadd 2014), and we support growing calls for a shift to holistic sex education within schools, characterised by an essentially positive approach towards sexuality, and an emphasis on the importance of positive healthy sexual development and general wellbeing, rather than on adolescent sexuality as morally problematic and potentially harmful (Bay-Cheng 2003; Ketting, Friele, and Micielsen 2015; Halpern 2010). It has been argued that a positive and holistic approach, focusing on emotional and physical pleasures of sex and relationships, should result in better outcomes for young people (McGeeney and Kehily 2016; Allen, Rasmussen, and Quinlivan 2014). School-based sexual health and relationships education needs to equip young people with knowledge and skills, both in the short-term and for later life (Hirst 2012). We suggest this is best delivered through mandatory provision of comprehensive, positive, inclusive, non-judgemental and skills-based learning for all young people.

Specific recommendations relating to content highlighted in our analysis include the provision of practical and realistic depictions of sexual activities and experiences; teaching about digital literacy and media/porn literacy to develop skills to navigate unregulated information sources effectively and to think critically about explicit material and portrayals of normative practices (Albury 2015; Elwick et al. 2013); the inclusion of activities to empower young people to understand and challenge gender norms, expectations and stereotyping (Banister and Begoray 2006); and, crucially, the development of communicative and critical skills to negotiate sexual interactions to enable the development of healthy, pleasurable and positive sexual relationships. Specific recommendations to improve delivery modes could include integrating online technologies in sex education delivery to maximise young people’s engagement and prepare them for effective, adaptive online self-teaching.

There are obstacles to implementing such provision in schools, and other learning contexts. Firstly, there are policy barriers. In Scotland, young people’s rights to comprehensive sexual health and relationships education are undermined by schools’ and local authorities’ inconsistent implementation of guidance, resulting in substantial variability in the standard and content of education delivered. In addition, there are strong socio-cultural barriers that influence learning about sexual health and relationships, especially in relation to gender and cultural norms, as highlighted by similarities in experiences across diverse age groups regarding accounts of experiences in school. While the effectiveness of population-level school-based sex education has been called into question (Elliott et al. 2013), new approaches rightly continue to be tested (Forsyth et al. 2018; Lohan et al. 2018). A lack of evidence of the effectiveness of school-based interventions may be due to ingrained cultural norms and the inherently slow-changing nature of these institutions, and of broader society (Couch et al. 2006). Sexual health and sexuality learning is bounded by a range of factors including social class, power, gender, and politics (Hirst 2012; Livingstone and Mason 2015; Ringrose and Barajas 2011). Fox and Bale (2018) argue that much of contemporary culture does not work to broaden sexualities, but in fact, restricts and promotes ‘a narrow and normative sexuality’ (p.394). Thus, transition to teaching more holistic diverse sexual health within schools, and to change norms around learning in other ways, will require a cultural shift. As such, Scottish Government’s decision to embed LGBTI rights-based teaching across the school curricula, and work on new comprehensive sexual health and relationships education resources for Scotland (co-produced with young people), is welcomed. Co-producing interventions with young people could increase their relevance and acceptability (Coll, O’Sullivan, and Enright 2018) and combining this with efforts to address and change socio-cultural norms is essential to provide the comprehensive, positive and inclusive learning that young people deserve.
